# The effectiveness of further education and training programs for plastic and aesthetic surgeons: an evaluation according to Kirkpatrick levels 1–3

**DOI:** 10.1186/s12909-025-07213-8

**Published:** 2025-04-30

**Authors:** Nora A. Hoppmann, E. H. Manassa, L. Kleining, J. P. Ehlers

**Affiliations:** 1 PAAU° Academy / bestofme GmbH, Ratingen, Germany; 2 Didactics and Educational Research in Healthcare, Medicine Department, Faculty of Health, Witten/Herdecke University, Witten, Germany

**Keywords:** Kirkpatrick model, Evaluation, Online training, Further education and training programme, Educational evaluation, Aesthetic plastic surgery, Educational success, Effectiveness measurement, Effectiveness analysis, Training evaluation

## Abstract

This article examines the success and effectiveness of a further education and training program on wrinkle injections for plastic and aesthetic surgeons using the Kirkpatrick model. Data on course satisfaction, learning progress and the application of what has been learnt in the professional environment were collected using two questionnaires before and after the wrinkle course. Log data from a learning management system is also used. Level 1 - reaction shows a high level of satisfaction with the further education and training program among course participants. Level 2 - learning shows a significant improvement after the wrinkle course when assessing the level of knowledge. Level 3 - behavior shows that the participants successfully apply the newly acquired wrinkle injection skills in their everyday work, especially when injecting with botulinum toxin A. The results of this study show that the Kirkpatrick model helps to measure the effectiveness and success of the offered wrinkle course. Important insights are gained for the design and evaluation of further education and training programs for plastic and aesthetic surgeons.

**Supplementary Information:**

The online version contains supplementary material available at 10.1186/s12909-025-07213-8.

## Introduction

Further education and training hold particular importance, especially in the medical context. These measures are intended to ensure that physicians demonstrate up-to-date expertise and field-specific competencies [1]. It’s important to distinguish between the terms “further education” and “training”. Further education involves acquiring additional knowledge, experiences, and skills. In the medical context, successful completion of such further education, for example, allows the designation as a specialist doctor. Training, on the other hand, occurs within the framework of the practiced medical profession and aims to maintain, expand, and adapt existing qualifications [2, 3]. Plastic-aesthetic surgery has emerged as a significant area of ​​surgery in recent decades, influencing not only the health but also the quality of life and confidence of patients. In this context, further education and training of professionals are crucial to ensuring that the latest techniques, procedures, and developments in this demanding medical field are applied. Working time restrictions, demographic changes, or medical-legal requirements present various challenges to treating physicians. Innovative further education and training programs play a crucial role in this regard. These programs help to expand knowledge, improve clinical skills, and enhance patient safety, making them a key element in quality assurance in plastic-aesthetic surgery [4, 5].

Online learning platforms enable physicians to access efficient, flexible, and location-independent further education and training opportunities, thus promoting professional development [6]. In addition to self-paced learning, digital learning platforms offer interactive learning methods, virtual simulations, and digital media. This flexibility allows medical professionals to integrate further education and training into their everyday work and immediately apply the acquired knowledge in patient care [7].

Further education and training programs pose certain challenges to both participants and trainers, as well as associated business partners. For participants, the primary focus lies not only on achieving clearly defined learning objectives but also on engaging with social aspects, such as the development of a community of practice. This community of individuals is characterized by a shared interest in specific topics as well as the exchange of ideas and insights. Thus, communities of practice provide an informal network primarily for knowledge, experience, and resource exchange [8]. Trainers of further education and training programs aim to measure and demonstrate the success of their training and offerings, for which Donald Kirkpatrick’s model offers a proven method of evaluation. Kirkpatrick’s model provides a structured and comprehensive approach to evaluating learning interventions, focusing on four different levels: Reaction, Learning, Behavior, and Results [9]. In the field of plastic-aesthetic surgery the Kirkpatrick model offers a holistic and practical evaluation of further education and training programs. The model examines not only the transfer of knowledge at the various levels, but also the acceptance and satisfaction of the course participants. Additionally, the study examines how effectively the acquired knowledge is transferred into practical application. Another advantage of the Kirkpatrick model is that it can be flexibly adapted to different specialist disciplines and training methods [10]. This evaluation encompasses both the theoretical foundations of the further education and training program and its practical applications in improving patient care.

By answering the following research questions important insights are gained, that contribute not only to scientific theory-building but also to practical implications for the design and implementation of further education and training measures. Within the framework of this article, important aspects of the evaluation of further education and training programs according to Kirkpatrick’s model at the Reaction, Learning, and Behavior levels are examined:

- How can learning platforms enhance engagement and learning success in further education and training programs?
- What individual and contextual factors influence knowledge acquisition?
- What methods promote workplace application of acquired skills?

The aim of this study is to evaluate the effectiveness of modern further education and training using the example of plastic-aesthetic surgery. In this context, the PAAU° Academy’s first wrinkle course is examined. This involves initially examining the reactions of participants to the further education and training, how knowledge acquisition develops, and whether the acquired knowledge finds practical application after the end of the intervention.

## Methods

To address the research questions, the Kirkpatrick model is complemented by a mixed-methods approach. This approach captures both quantitative improvements in knowledge and qualitative insights into participants’ experiences. This ensures a comprehensive evaluation of the further education and training program. The Kirkpatrick model consists of four levels of evaluation: reaction, learning, behavior, and results. Each level examines different aspects of further education and training, enabling a comprehensive assessment of their effectiveness. The model is also applicable to other areas, including the measurement of business success. According to Kirkpatrick [11], the evaluation of further education and training should not only consider on participants’ reactions but also consider acquired knowledge, application in practice, and long-term results. The Kirkpatrick model has established itself as one of the leading methods for evaluating further education and training programs [12]. It provides firstly a structured and systematic approach for evaluating such training programs. Secondly, it emphasizes outcome-oriented evaluation by considering the actual performance of course participants and the broader impact on associated organizations. Strengths and weaknesses are identified at each level of the model, enabling organizations to improve further education and training programs accordingly [13]. In this initial investigation, the first three levels of the Kirkpatrick model are examined: reaction, learning, and behavior. The result level is considered separately, because the effects of the further education and training program on the organization (e.g., increased awareness, improved patient care, monetary results) only manifest with a longer time lag. Therefore, immediate measurement following the training program is not appropriate. Moreover, the result level relates primarily to management and organizational level. While the other three levels focus on participants of the training program. In this further investigation, expert interviews will be conducted with representatives from the management of participating companies and the professional association. These interviews will be analyzed using qualitative content analysis according to Mayring [14]. In this study, the Kirkpatrick model is applied as follows:

1. Reaction level
  In questionnaires specifically designed for the study, participants’ satisfaction is assessed as part of a pre- and post-test. They provide their evaluations regarding satisfaction, the usefulness of the content, and their experiences with further education and training measures. The collected feedback is used to immediately optimize the training program after course completion. In addition, log data from the learning management system is analyzed.
2. Learning level
  In questionnaires specifically developed for this study, participants assess their knowledge related to the primary learning objectives of the wrinkle course as part of a pre-test and post-test. This survey take place before and after the further education and training program. The resulting data are analyzed to evaluate the knowledge and skills acquired throughout the course. In addition, log data from the learning management system are examined.
3. Behavior level
  In questionnaires specifically designed for the study, the post-test assesses the transfer of acquired knowledge into practical application. In particular, it examines whether participants have performed injections with hyaluronic acid or botulinum toxin A.
4. Results level
  To measure the long-term outcomes of the further education and training program, a separate study is conducted.

An ethics application was submitted to the responsible ethics committee at Witten/Herdecke University before the start of the study. The positive ethics vote can be found under file number S-208/2023. Before course participation, all respondents provided written informed consent, following General Data Protection Regulation (GDPR) guidelines. A declaration of consent was provided to participants, assuring them that data analysis would be fully anonymized as part of a dissertation. This applies to the log files from the learning management system and the pre- and post-test. Data collected from both questionnaires were exported anonymously using CSV exports from the Moodle learning management system and LimeSurvey software. To ensure the transparency and scientific traceability of this evaluation, the SRQR checklist was used as a guideline for reporting. The SRQR criteria ensure a structured presentation of the research processes and support the methodological quality of this study [15] (Appendix C: SRQR-Checklist).

**Fig. 1:**
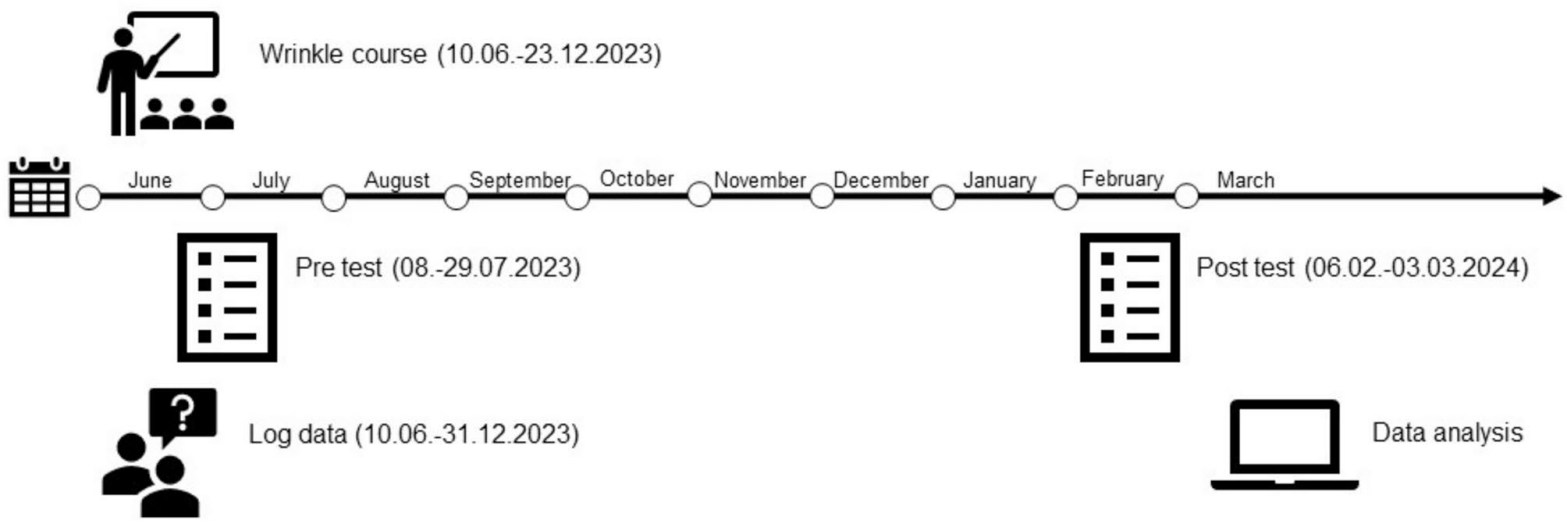
Timeline for conducting the study (own illustration)

The measurement of learning success focuses on the first course on “Wrinkle Treatment with Botulinum Toxin and Fillers” (wrinkle course) offered by the PAAU° Academy/bestofme GmbH (Fig. 1). The wrinkle course runs from 10th June to 23rd December 2023 and consists of 14 thematically aligned online sessions. These sessions take place every two weeks on Saturdays from 10:00 am to 11:30 am. Between each session, participants complete assigned tasks through self-study. These self-study tasks must to be completed by Thursday evening prior to the next online meeting. They are submitted digitally for evaluation via the Moodle learning management platform. Additionally, on 10th June 2023, a digital technical introductory course on the software is conducted. The wrinkle course begins with a group of 28 participants, of whom 27 complete it. In accordance with the study’s design, all individuals enrolled in the course are invited to participate at the pre-test. After completing the course, they are contacted again to take part in the post-test. As a result, a separate selection procedure is not required.

**Fig. 2:**
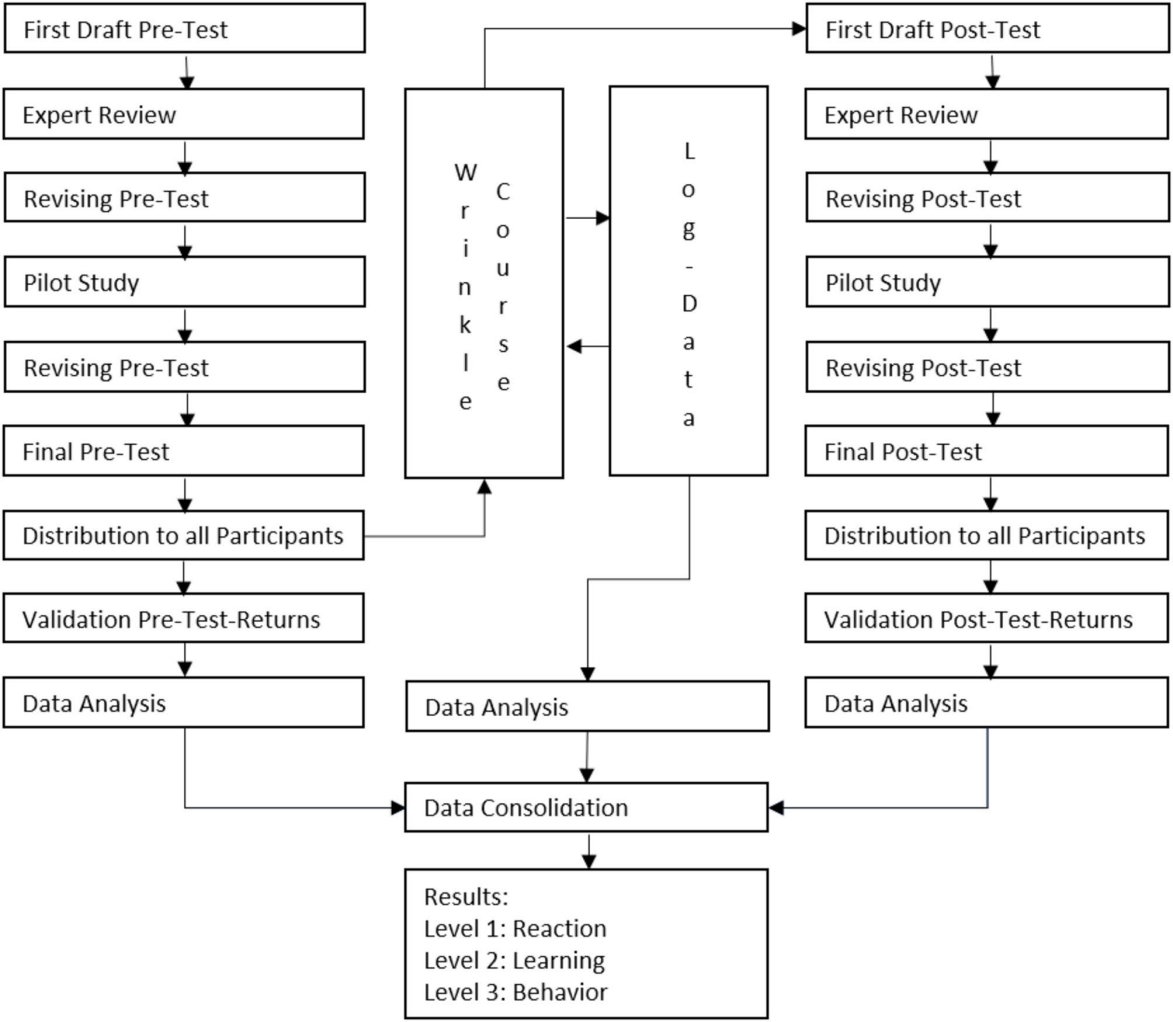
Flowchart of study methodology (own illustration)

The study employs a quantitative research approach (Fig. 2). To this end, a questionnaire for the pre-test is developed and made available digitally to participants via the Moodle learning management system at the beginning of the wrinkle course. During the online meeting on 8th July 2023, participants are informed about the evaluation, and the questionnaire is published. Participation in the pre-test remains open until 29th July 2023 and targets all participants of the wrinkle course. The questionnaire consists of four sections containing 28 items. The first section includes socio-demographic questions and inquiries about the participants’ professional background. The second section explores participants’ expectations of the wrinkle course, using open-ended questions to allow for free expression of opinions. The third section aims to assess participants’ current knowledge of various learning objectives covered in the course. These learning objectives are derived from the curriculum of the training program. The fourth section focuses on participants’ personal experiences with training programs. It gathers information on the number of training programs attended annually, previous experience with digital training programs, and decision factors for attending training programs. Responses are collected using a four-point Likert scale ranging from “very important” to “unimportant” (Appendix A: Pre-test questionnaire).

Building upon this, a second questionnaire is designed and provided to participants six weeks after the end of the training program. The questionnaire, hosted on the LimeSurvey platform at the University of Witten/Herdecke, is available from 6th February to 3rd March 2024. Participants are contacted via email and directed to the online questionnaire through a link. The questionnaire consists of three sections containing 30 items. As in the pre-test, the first section collects socio-demographic information and professional background. The second section assesses participants’ satisfaction with the wrinkle course, using a six-point Likert scale. It also includes questions about how well the skills learned in the training program transfer to practice. The third section evaluates participants’ knowledge in various medical areas aligned with the training program’s learning objectives. Again, a symmetrical six-point response scale is used (Appendix B: Post-test questionnaire).

To ensure validity, the pre- and post-test questionnaires undergo expert review. This process includes verifying that the questionnaires cover all relevant aspects of the study. The expert review also examines whether the questions are meaningful, understandable, and complete. Based on the results of the expert review, the questionnaires are revised and tested on a group of three people in a pilot study to ensure clarity before full implementation [16]. The pre-test and post-test questionnaires are specifically developed for this study and have not been published elsewhere (Appendices A & B).

The collected data from both questionnaires is checked for completeness and analyzed using Microsoft Excel statistical program. Since all questionnaires are fully completed, data analysis begins immediately afterward [17]. Initially, the results of the pre-test are evaluated independently, followed by the analysis of the post-tests results. Subsequently, the findings from both surveys are compared and analyzed together. Descriptive and exploratory statistics are used to identify irregularities and notable patterns in the collected data [18, 19]. Especially when assessing learning outcomes, the analysis allows conclusions to be drawn about the effectiveness of the training program. Descriptive statistics provide an initial overview of the data by calculating means, standard deviations, and medians to determine central tendencies and variability in the responses [20]. Cohen’s d is used as a metric to categorize learning gains. It helps determine the strength of the difference in the arithmetic means between two samples. Due to the different sample sizes (Pre-Test *n* = 18; Post-Test *n* = 19), the pooled standard deviation is applied in the calculation [21]. Exploratory analysis, including factor analysis, examines the underlying structure of relationships between variables, identifying patterns or commonalities in participants’ responses [22]. The identified values are used to create diagrams and graphs. Correlation analysis visualizes the data and illustrates the distribution and relationship between variables [23]. Responses to open-ended questions are analyzed using Mayring´s content analysis, with answers grouped into thematic clusters and weighted according to their frequency of occurrence [14, 24].

In addition to data collected from the questionnaires, which reflect the subjective assessment of the respondents, data from the Moodle learning management system is analyzed. Log data from the platform provides an objective measure of user behavior. By combining both data sources, a more comprehensive picture of learning effectiveness emerges. This enables a more accurate evaluation and targeted optimization of the further education and training program [25]. The analyzed data consists of system-recorded log entries from 10th June to 31st December 2023. Initially, the log data is cleaned to include only participant-related entries and is anonymized to prevent identification of individuals. In total 27,346 records are collected and processed. Microsoft Excel is used to analyze the log data. A pivot table is applied to structure the data, and events are clustered. These events represent actions taken by participants on the learning management platform, such as sending messages, accessing learning materials, or submitting self-study assignments [19]. A total of 63 distinct events is identified. Each event is assigned a corresponding number of recorded actions. For example, the event “Dashboard displayed” includes 1,753 actions. It indicates that the dashboard is accessed 1,753 times during the data collection period. Additionally, self-study completion and awarding of badges are examined in more detail. Self-study data is analyzed for each session of the wrinkle course. It considers when reminders are sent to participants and when tasks are completed. The data is categorized as either “Completed before reminder” or “Completed after reminder.” Similarly, badge awards are analyzed for each session. The resulting data is visualized through graphical representations to highlight trends and developments over time. A separate analysis is conducted for data classified under the category “Community of Practice,” which describes the extent to which participants form a knowledge-sharing community during the training program. Events within this category are assignable to both level 1 and level 2 of the Kirkpatrick Model.

All results from the questionnaires and log data are mapped onto the Kirkpatrick model levels. The results are assigned to either level 1: participants’ reaction to the training program, level 2: learning outcomes and knowledge acquisition, or level 3: behavioral changes resulting from the training program.

## Results

The study records a response rate of 64% (18 out of 28) for the pre-test and 68% (19 out of 28) for the post-test (Table 1). The average age of the respondents is 35.3 years in the pre-test and 36.4 years in the post-test. In the pre-test, participants indicate their professional roles as follows: 44% assistant physicians, 22% physicians, 17% specialist physicians, 11% senior physicians, and 6% training assistants. In the post-test, the distribution shifts to: 26% assistant physicians, 37% physicians, 26% specialist physicians and 11% senior physicians. This shift in roles suggests that after completing the wrinkle course, the proportion of assistant physicians decreases, while the proportions of physicians and specialists increase. The percentage of senior physicians remains unchanged. On average, participants report having attended 17.72 training or further education events prior to the wrinkle course. This indicates that, before taking part in the current program, they had completed nearly 18 training sessions on average.

**Table 1:** Characteristics of wrinkle course participants (own presentation)

	Pre-test^a^	Post-test^b^
**Age**		
30–35	12	11
36–40	3	5
40–45	2	2
46–50	0	0
50–55	1	1
**Professional roles**		
Assistant physician	8	5
Senior physician	2	2
Physician	4	7
Specialist physician	3	5
Training assistant	1	0
**Number of further education and training programs**		
0–10	11	
11–20	2	
21–30	3	
31–40	0	
41–50	1	
over 50	1	

^*a*^*n = 18;*^*b*^*n* = 19

In addition, participants are asked to describe in free-text responses what content a wrinkle course should cover and how important the formation of a community of practice is to them. Since the question format allows for open responses, the answers are analyzed using content analysis and ranked according to frequency. The most frequently mentioned categories are: indication (9 mentions or 50%), treatment planning (8 mentions or 44%), material science (8 mentions or 44%), anatomy (7 mentions or 39%) and pitfalls (7 mentions or 39%). The relevance of forming a community of practice is rated as very important by four participants, as important by eight participants, as moderately important by three participants, as less important by two participants, and as not important by one participant. A total of four free-text responses indicate that the formation of a community of practice is sometimes a new experience for the participants or that the realization of such a community has not been successful so far.

In the final part of the questionnaire, participants are asked about their previous experiences with further education and training programs, as well as the criteria for selecting a particular program. A total of 39% of participants state that they attend 1–2 training courses per year. Another 44% attend 3–5 training courses per year, and 17% participate in more than 5 training courses annually. Regarding previous experience with online courses, 72% of participants have already completed a digital course program. The results regarding the criteria for selecting a specific training program show that practice-related topics are considered very important (89%) or important (11%) by the participants. In contrast, support from the employer is considered very important by only 6% and important by 39%. As a selection criterion for a specific further education and training program, face-to-face events are deemed unimportant by 6% and less important by an additional 39%.

With the help of these results, the respondents are characterized, providing insight into which participants took part in the survey. The other collected results are assigned to a specific level according to the Kirkpatrick model.

The second questionnaire, deals, among other things, with satisfaction (Table 2). The results are assigned to level 1: participants’ reaction to the training program. The distribution of satisfaction across the key areas is as follows:

**Table 2:** Distribution of satisfaction among the focal points (rounded to full percentage points) (own presentation)

Focal points	extremely satisfied	very satisfied	little satisfied	little dissatisfied	very dissatisfied	extremely dissatisfied
Wrinkle course overall	29%	76%	0%	6%	0%	0%
Course content	21%	63%	11%	5%	0%	0%
Course structure	21%	47%	26%	5%	0%	0%
Frequency online meetings	37%	58%	0%	5%	0%	0%
Course material	16%	58%	26%	0%	0%	0%
Learning platform - learning support	26%	42%	26%	5%	0%	0%
Learning platform - creating a community of practice	0%	47%	37%	16%	0%	0%
Learning progress after course end	11%	68%	16%	5%	0%	0%

*n* = 19

The results show that 29% of participants are extremely satisfied, and 76% are very satisfied with the wrinkle course. Only 6% are a little dissatisfied. The frequency of the online meetings is rated as extremely satisfied by 37% of respondents and very satisfied by 58%. The length of the wrinkle course is considered too long by 16% of participants, just right by 74% and too short by 11%. Furthermore, 68% of participants feel that a community of practice has formed after the end of the course, while 32% state that no community of practice has formed.

Furthermore, log data collected from the learning management system provides additional insights how participants react to the wrinkle course. From the 27,346 processed data records, a total of 1,573 logins to the learning management platform are recorded. In relation to the 28 participants, each participant logs into the platform around 56 times. A total of five sections must be completed during the course. These sections are accessed a total of 3,569 times. As part of the self-study program, tasks must be completed between the online meetings. Additional tasks can be undertaken in the form of deep dives to expand knowledge. A total of 1,222 activities are completed. A total of 5,666 activities are accessed by participants during the wrinkle course. Participants are awarded a badge for special achievements. In the performance category, which tracks attendance and participation during the online meetings, a total of 254 badges are awarded over the entire duration of the wrinkle course. Additionally, a badge is awarded for participation in self-study, with a total of 218 badges given. The participation in all completed self-study activities is also analyzed. It shows that participation in the self-study tasks for the first six sessions ranges between 80% and 93%. For sessions 7 to 12, participation ranges between 32% and 77%. For the last two sessions (sessions 13 and 14), no self-study tasks are given. The timing of self-study task completion by the participants is also analyzed. After each session, participants have 12 days to complete the tasks. After 10 days, a reminder is sent to participants via email, reminding them of the tasks and the end date. The following results are obtained in absolute figures:

**Fig. 3:**
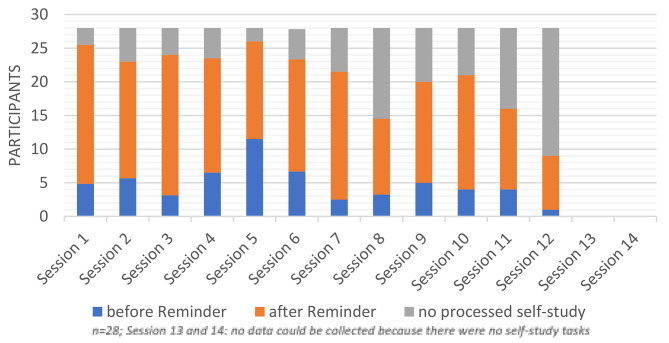
Average self-study completed before and after reminder by session (own illustration)

The chart (Fig. 3) shows that the majority of participants complete the self-study tasks only after the reminder is sent. Looking at the first session, it can be seen that 5 participants had already completed the tasks before the reminder was sent, but 20 participants only became active after the reminder was sent. These results are assigned to level 1: participants’ reaction to the further education and training program.

By surveying the level of knowledge at the beginning (using the pre-test) and six weeks after the end (using the post-test) of the wrinkle course, it is possible to establish comparative values. These results are assigned to level 2: learning outcomes and knowledge gain through the training program. For this purpose, average values are calculated from the participants’ assessments for each category and compared. Additionally, values for pooled standard deviation and Cohen´s d are calculated. The results indicate that the participants have the greatest increase in knowledge after the end of the wrinkle course in the areas of treatment plan preparation, injection system, injection technique, and complication management. Prior to the wrinkle course, the participants assess their level of knowledge in these areas ranging from satisfactory to sufficient. After the course, the level of knowledge is assessed as good. The values for the individual learning objectives are presented below. Cohen´s d analysis reveals a strong effect in 10 out of 13 categories (Cohen´s d > 0.8). In the “Aesthetics” category, a medium effect is observed (Cohen´s d between 0.5 and < 0.8). A low effect is found in the “Patient communication” category (Cohen´s d between 0.2 and < 0.5) and no or very little effect is recorded in the “Legal aspects and hygiene” category (Cohen´s d between 0.0 and < 0.2) (Table 3).

**Table 3:** Average values for the level of knowledge before and after the wrinkle course, pooled standard deviation and Cohen´s d (own presentation)

Learning objective	Average value			
	Before course start^a^	After course end^b^	Pooled Standard deviation	Cohen´s d
Facial anatomy	2.83	2.11	0.76	0.96
Aesthetics	2.67	2.05	0.88	0.70
Facial ageing	2.94	1.89	0.65	1.62
Legal aspects and hygiene	2.83	2.74	1.20	0.08
Patient information and documentation	2.50	1.32	0.73	1.62
Patient communication	2.17	2.32	0.78	0,48
Preparation treatment plan	3.72	1.74	0.91	2.17
Risks associated with wrinkle injections	3.06	1.68	0.70	1.96
Selection of suitable injection products (Botulinum toxin A or hyaluronic acid)	3.22	2.16	0.95	1.12
Indication and contraindication	3.33	2.00	0.86	1.55
Injection system and injection technique	4.11	2.11	0.89	2.26
Complication management	4.22	2.53	0.89	1.90
Pre- and aftercare	3.61	2.16	0.91	1.60
TOTAL average value	3.17	2.06	0.85	1.31

^*a*^ *n=18;* ^*b*^ *n=19*

^*a; b*,^ *1.0 = very good; 2.0 = good; 3.0 = satisfactory; 4.0 = sufficient; 5.0 = poor; 6.0 = very poor*

The log data indicates, in the area of learning outcomes and knowledge gain, that a total of 69 posts are created by participants in forums for exchange. These posts include questions to the course group, such as inquiries about injection techniques or special cases. As part of the self-study program, a total of 174 submissions are created, and 71 responses are provided in form of free-text answers to address questions. Additionally, 81 files are uploaded, which are created as part of a photo assignment.

For the deep dive test, participants were allowed an unlimited number of attempts to complete the associated task. The prerequisite is that all questions must be answered correctly. In total, the deep dive test was accessed 768 times. Of these, 291 tests were submitted for assessment. A badge was awarded 282 times, as these tests met the required criteria. These results are assigned to level 2: learning outcomes and knowledge gain through the further education and training program.

The development of the Community of Practice is reflected in the communication and interaction activities on the learning platform. A total of 159 private messages are exchanged, including messages between participants as well as those directed to instructors. Additionally, 63 messages are sent via the group chat, which includes all participants and instructors of the wrinkle course. In total, 2,814 messages are accessed by participants, indicating a high level of engagement with the communication tools. Within the course forums, 1,909 messages are posted. These forums serve as a space for both professional exchange and general inquiries. User profiles are accessed 336 times, although it is not possible to distinguish whether these views pertain to one’s own profile or that of another participant. Profile information is created or updated 99 times, following the request made at the beginning of the course for participants to complete their profiles and upload a photo. Moreover, 17 badges are given to participants for providing documentation for the entire wrinkle course. These contributions are significant studies or scientific articles that were of particular interest for the course. These badges are categorized as Community of Practice. These results can be assigned to both level 1: participants’ reaction to the further education and training program and level 2: learning outcomes and knowledge gain through further education and training program.

The second questionnaire assesses the transfer of acquired knowledge into clinical practice, corresponding to level 3: changes in participants’ behavior within their professional environment as a result of the training program. A key item investigates whether participants had performed wrinkle injections following the completion of the course.

Within six weeks after the conclusion of the wrinkle course, 74% (14 out of 19) of the participants report having already performed wrinkle injections using botulinum toxin A. All individuals who have conducted injections by this time indicate that the course increased their confidence in administering injections. Approximately 25% of respondents emphasize the relevance of the anatomical knowledge and theoretical principles conveyed during the course. Furthermore, one-third of the participants report an enhanced understanding of injection techniques and injection points, which contributes to their increased confidence when performing injections. Participants also note that the wrinkle course reduces psychological barriers to performing injections and facilitates treatment planning.

Injections with dermal fillers, such as hyaluronic acid, are performed by 21% (4 out of 19) of wrinkle course participants within six weeks following course completion. All participants who have administered filler injections at this stage report that the course contributed to an increased sense of confidence regarding injection procedures. To explain this increased confidence, 75% of these participants indicate that the wrinkle course provided a strong theoretical foundation, particularly in areas such as facial anatomy. Additionally, one-third of respondents highlight the benefit of learning about specific treatment techniques, which they report as facilitating the initial implementation of filler injections in practice.

The findings of this study reveal a positive correlation between participants’ satisfaction with the course (level 1) and their perceived learning gains (level 2). High levels of satisfaction regarding the overall course experience, including the content, structure, learning management system, and subjective knowledge acquisition, are reflected in the reported learning outcomes. Participants report a perceived learning gain in 11 out of 13 defined learning objectives. Furthermore, behavioral changes (level 3) are evident, particularly in the practical application of botulinum toxin A injections, suggesting that the course effectively supports the transfer of theoretical knowledge into clinical practice. The comparatively lower use of dermal fillers may indicate a heightened awareness of potential complications, likely fostered by the course content. This suggests a reflective and risk-aware approach to clinical decision-making among participants.

## Discussion

At the first level of the Kirkpatrick model, the reaction of participants to the further education and training program are assessed. Participants report high levels of satisfaction with the wrinkle course, as evidenced by questionnaire feedback. They express positive evaluations regarding the course content, structure, learning platform for instructional support, learning progress, and materials provided upon course completion. Log data from the learning management system further indicate consistent engagement with the platform, with each participant accessing the system approximately twice per week throughout the duration of the wrinkle course. Self-directed study is also received positively, particularly during the initial phase of the course [26]. However, it becomes apparent that most participants complete self-study assignments only after receiving written reminders. This observation does not conclusively determine whether reminders serve as the primary motivator or whether participants intentionally schedule their study time closer to the deadline. One possible explanation is that participants may not consistently monitor deadlines or proactively plan their learning activities [27].

The positive reaction of participants to the wrinkle course can be linked to the bandwagon effect through the formation of a community of practice. This describes the fact that ideas, suggestions or points of view are given disproportionate consideration in groups and can therefore influence the objectivity of the group decision [28]. A positive atmosphere among participants can therefore trigger positive opinion-forming. In this context, providers of further education and training programs should be aware of the impact of group effects. Both positive and negative individual voices can thus be given more space [28].

The findings indicate that participants respond very positively to the wrinkle course. Both the course and its content are perceived as positive and expectations are met. Consequently, it can be concluded that learning platforms provide an effective means of enhancing participants’ reactions to continuing education and training programs [29, 30]. Learning platforms support and motivate learners throughout the educational process and contribute to the achievement of learning outcomes [28]. By integrating varied self-study tasks such as interactive learning activities or peer reviews, participants can actively participate in their learning process and support each other. A personal dashboard and a progress indicator enable a personalized learning experience. In addition, forums offer the opportunity to share experiences and the chat function allows participants to exchange personal experiences. These elements promote learner engagement and increase motivation to consolidate and apply acquired knowledge [31,32] The high satisfaction reported at Level 1 of the Kirkpatrick model demonstrates that e-learning formats are well-received, particularly when compared to traditional face-to-face training. This trend is further supported by current developments in medical education, where digital platforms are increasingly adopted as flexible and cost-effective alternatives [33, 34]. The integration of videos on injection techniques and the use of interactive elements motivates learners to spend time in the learning management system and at the same time promotes understanding of the practical implementation [35]. As the initial stage of the Kirkpatrick model, Level 1 provides critical insight into the program’s perceived effectiveness and serves as a foundation for identifying areas for improvement.

In the second level of the Kirkpatrick model, participants´ learning outcomes are assessed. The average test results demonstrate a significant improvement in the knowledge acquisition following completion of the wrinkle course in 11 out of 13 categories. These assessments can be attributed to the Hawthorne effect. It refers to individuals’ tendency to alter or enhance their behavior when they are aware of being observed in a study. Consequently, the increased knowledge observed after the course may reflect an elevation in performance due to this effect. Additionally, the John Henry effect, in which a person makes a special effort or performs at an above-average level in order to achieve a certain goal, is also conceivable [36]. Interestingly, in one specific category, participants rate their knowledge higher before the course than after. This finding is likely attributable to the Dunning-Kruger effect, a cognitive bias in which individuals with limited knowledge overestimate their expertise. As participants’ knowledge increases, they become more aware of the complexity of the subject matter and are thus able to assess their competencies more realistically, sometimes even rating their post-course knowledge lower than before [37]. These findings are particularly relevant in aesthetic plastic surgery, where theoretical knowledge, such as anatomy, injection techniques, and material science, provides a crucial basis for practical skills [33]. Beyond test results, data from the learning platform logs reveal that participants actively engaged with one another via forums and chat functions. They also updated their profiles and showed notable interest in the profiles of fellow learners. These behaviors reflect a high level of engagement and support the conclusion that participants achieved meaningful learning outcomes and significantly enhanced their knowledge through the course [38]. In principle, the acquisition of new knowledge and skills is influenced by a variety of individual and contextual factors. These include personal learning styles, prior knowledge, motivation, learning environment and the work context. To maximize learning success, further education and training programs should take these factors into account and design them accordingly. The use of different learning activities on a learning platform ensures variety in learning and motivates at the same time [39]. Additionally, self-study materials should be adapted to accommodate different learning preferences. With the help of peer learning, learners can work together and support each other. The focus is on the exchange of knowledge, experiences and perspectives [40]. Learning management systems offer flexibility and opportunities for individualization, especially for self-study. In this context, a blended learning approach is particularly beneficial. In aesthetic plastic surgery, combining digital learning environments for theoretical content with hands-on practical training allows learners to benefit from both knowledge growth and the development of a professional community [41, 42]. Overall, Level 2 of the Kirkpatrick model serves as a crucial step in evaluating the effectiveness of the training program. The data indicate that the intended learning objectives are successfully met by the participants.

In the third level of the Kirkpatrick model, behavioral change is assessed by measuring the extent to which participants implement the knowledge, skills and attitudes acquired in the training program in their working environment. It focuses on whether the training leads to concrete changes in participants’ behavior, thereby facilitating the transfer of learning into practical application. According to Kirkpatrick, several conditions must be met for behavioral change to occur: participants must have the motivation to change, they must understand how to implement the change, the work environment must foster a supportive atmosphere, and there must be a reward or recognition associated with the change process [43]. However, various barriers can hinder the transfer of learning into behavior. These include limited opportunities to apply the newly acquired knowledge, a lack of conviction that the effort will change performance, resistance from managers, and a lack of support or resistance from colleagues when applying new approaches [43].

The results of the post-test indicate that 74% of respondents have already carried out wrinkle injections with botulinum toxin A themselves after completing the wrinkle course. This is accompanied by the fact that all respondents stated that the wrinkle course has helped them to carry out injections with botulinum toxin A. It can therefore be concluded that the majority of participants actively applied the knowledge on wrinkle injections with botulinum toxin A acquired in the wrinkle course. In contrast, only 21% of respondents have performed injections with dermal fillers such as hyaluronic acid after the end of the wrinkle course. Here too, all respondents stated that the wrinkle course has helped them to carry out injections with dermal fillers.

There are several reasons why injections with botulinum toxin A are more common than those with dermal fillers such as hyaluronic acid following the wrinkle course. First, injections with botulinum toxin A are associated with fewer complications, and when complications do occur, they tend to be less severe compared to those involving hyaluronic acid [44–46]. Botulinum toxin A interrupts the transmission of impulses from the nerve to the muscle, causing the muscle to relax for a certain period of time. The effect builds up slowly after the injection and the first results are seen after 24–72 h. The treatment lasts for three to six months and depends on the dosage and the skin area. Possible areas of application in addition to wrinkle injections include the treatment of excessive sweating and migraines [47]. Hyaluronic acid, on the other hand, shapes and builds up volume under the skin, making the skin appear plumper [48]. This can result in visible aesthetic changes for patients. The practitioner must therefore be very familiar with the use of hyaluronic acid and its dosage so that no undesirable effects occur. Overall, the areas of application for botulinum toxin A are therefore wider than for hyaluronic acid. Another advantage of using botulinum toxin A is that it is completely reversible without any additional measures, as it is broken down by the body over time [44]. In the case of hyaluronic acid, reversal may require active measures such as the administration of hyaluronidase [45].

To summarize, treatment with botulinum toxin A is generally easier to perform and is associated with a lower risk of complications. This may explain why fewer treatments using hyaluronic acid are carried out following completion of the training program. Additionally, the wrinkle course may enhance participants’ awareness regarding the use of both botulinum toxin A and hyaluronic acid. By providing comprehensive information on injection techniques, materials, risks and side effects, the course fosters a solid understanding of wrinkle treatment practices [49]. However, the survey does not provide any information as to whether the participants actually had the opportunity to perform injections with botulinum toxin A or hyaluronic acid in their everyday work. Possible reasons for this could be that no test subjects are available or that the participants themselves do not yet have their own practice or practice rooms with the possibility of injecting wrinkles.

These results indicate that the participants actively apply the majority of what they learn during the wrinkle course in practice. In principle, various methods and measures should be used to apply the knowledge learnt from the wrinkle course in the workplace. These include, in particular, case studies, practical exercises, coaching, feedback mechanisms and the integration of learning content into everyday working life [13]. Guided hands-on courses, for example, can help participants overcome initial barriers to performing wrinkle injections. Such formats also ensure that learners have the opportunity to practice injection techniques in a controlled and supportive setting. Depending on the working environment of the participants, it may not always be possible to carry out wrinkle injections in their everyday work. Level 3 of the Kirkpatrick model is crucial to ensure that the further education and training program not only imparts theoretical knowledge, but also has a measurable impact on the participants’ working behavior in practice.

In order to ensure positive results from the wrinkle course in long term, various didactic approaches should be pursued. On the one hand, a high use of interactive and practical learning methods, such as case studies, learning videos and group work. In addition, blended learning approaches can make it easier to put theoretical knowledge into practice and give course participants more confidence in guided wrinkle injections. The combination of self-study and online meetings offers participants flexibility and can therefore promote the long-term acquisition of knowledge. Learning progress can be monitored and secured through learning checks in the form of tasks [50].

Despite the comprehensive application of Kirkpatrick’s model for the evaluation of further education and training programs in plastic and aesthetic surgery, certain limitations must be considered that may restrict the interpretation of the results. One major limitation is the complexity and subjectivity of the assessment at the behavioral change (level 3) of Kirkpatrick’s model. The implementation of newly acquired skills and behaviors in the workplace is often dependent on a variety of factors, including individual motivation, organizational support and clinical experience [43]. As the assessment at this level is based on self-report, biases or uncertainties may occur that could affect the reliability of the results. Furthermore, the generalizability of the results may be limited as the study is restricted to a specific group of plastic and aesthetic surgeons and to a specific training program. Differences in clinical practice, level of training and regional circumstances could lead to variable results that may not be transferable to other contexts. The relatively small sample size (*n* = 27) limits the generalizability of the results. Moreover, the long-term retention of knowledge was not assessed, which substantiates the necessity for further longitudinal studies.

## Conclusion and prospects

This study on the evaluation of a wrinkle course using the Kirkpatrick model provides important insights into the effectiveness and benefits of further education and training programs in plastic and aesthetic surgery. By systematically analyzing the various levels of the Kirkpatrick model, the study generates multidimensional findings that contribute both to the advancement of academic theory and to practical improvements in clinical practice.

At the first level of the Kirkpatrick model, the reaction level, participants’ responses to the wrinkle course are overwhelmingly positive. High levels of satisfaction are particularly evident in evaluations of the course structure, the learning content, and the provided materials. Log data reveal that participants actively engage with the learning platform and consistently complete the self-study tasks. Additionally, high levels of interaction in forums and chats suggest the emerge of a community of practice. These findings indicate that the wrinkle course is well received and meets or exceeds participants’ expectations. This is an important starting point for the further evaluation of the effectiveness of the further education and training programs.

The second level of the Kirkpatrick model, the learning level, also yields positive results regarding participants’ acquisition of knowledge and skills. The findings indicate that the learning content is effectively absorbed and understood. The defined learning objectives are successfully achieved. These outcomes demonstrate the efficacy of both the learning materials and the course design as a whole. The wrinkle course helped to improve expertise and clinical skills in the field of wrinkle injections. This contributes to better preparation for a broad spectrum of wrinkle treatments and the fulfilment of patient needs.

The third level of Kirkpatrick’s model, the behavioral level, yields mixed results. While many participants report successfully integrating newly acquired knowledge into their clinical practice particularly in the application of botulinum toxin A, challenges arise in the implementation of dermal filler techniques, such as those involving hyaluronic acid. Factors such as individual motivation, organizational support, and the clinical work environment play a critical role in determining the extent to which new skills and behaviors are adopted in practice.

This study focuses on the first three levels of the Kirkpatrick model. The fourth level of the Kirkpatrick Model: Results will be explored by examining the positive outcomes identified in this study, which are primarily based on participants’ subjective evaluations. The main aim is to assess the impact of the finding of levels 1–3 on the business performance of participating companies and the professional association. The future study of level 4 will therefore determine the extent to which the acquired knowledge and its practical application contribute to sustainable improvements in organizational outcomes. Ultimately, this will enable a comprehensive and holistic evaluation of the wrinkle course.

## Electronic supplementary material

Below is the link to the electronic supplementary material.

Supplementary Material 1

Supplementary Material 2

Supplementary Material 3

## Data Availability

The data sets available during the current study and/or the analyzed data sets can be requested from the corresponding author upon reasoned request.

## Declarations

### Clinical trial number

A clinical trial number is not applicable.
