# Supplementary material for: The effectiveness of further education and training programs for plastic and aesthetic surgeons: an evaluation according to Kirkpatrick levels 1–3
Source: BMC Med Educ. 2025 Apr 30;25:636. doi: 10.1186/s12909-025-07213-8 (PMC12042626; doi:10.1186/s12909-025-07213-8)
Supplement: Supplementary file 1 — Supplementary Material 1 [file 12909_2025_7213_MOESM1_ESM.pdf]

Appendix A: Pre-test questionnaire

Pre-test questionnaire for the further development and improvement of further education and training programs for specialists in plastic-aesthetic surgery

Socio-demographic issues and occupational characteristics

- Age in years _______ *(free text)*
- Gender *(Single Choice)*

  O female

  O male

  O divers

- Activity performed ____________ (free text)
- Number of further education and training courses to date _______ *(free text)*

Expectations for the Wrinkle Course

- Please briefly explain in keywords what you think a wrinkle course should convey?

  __________________________ *(free text)*

- How important is the formation of a community of practice that continues to exist after the end of the course?

  __________________________ *(free text)*

Questions about the learning objectives

- Please indicate how you assess your level of knowledge on the following topics (very good (1); good (2); satisfactory (3); sufficient (4); poor/poor (5); inadequate/very bad (6)):

	(1)	(2)	(3)	(4)	(5)	(6)
Anatomy of the face						
Aesthetics of a beautiful and natural face						
Facial aging						
Legal aspects, hygiene and liability for medical treatment						
Education and documentation (incl. photo documentation) of patients						
Patient communication						
Preparation of a treatment plan						
Risks of wrinkle injections						
Selection of suitable injection products (botulinum toxin type A or hyaluronic acid)						

Appendix A: Pre-test questionnaire

Indication and contraindication of wrinkle treatments						
Injection system and injection technique for the injection of wrinkles						
Complication management in wrinkle treatment						
Pre- and aftercare for wrinkle treatment						

Personal experience with continuing education and training courses

- How often do you take part in job-specific further education and training? *(Single Choice)*

  O Less than 1x per year

  O 1-2x per year

  O 3-5x per year

  O more than 5x per year

  O Never

- Have you already taken part in an online course, e.g. as part of further education or training? *(Single Choice)*

  O yes

  O no

- How important are the following factors to you when you decide on further education or training?

	unimportant	less important	important	Very important
Part-time offer				
Practical topics				
Support from employers				
Availability of learning materials				
Individual learning and working pace				
Digital course availability				
Face-to-face events				
