# Supplementary material for: The effectiveness of further education and training programs for plastic and aesthetic surgeons: an evaluation according to Kirkpatrick levels 1–3
Source: BMC Med Educ. 2025 Apr 30;25:636. doi: 10.1186/s12909-025-07213-8 (PMC12042626; doi:10.1186/s12909-025-07213-8)
Supplement: Supplementary file 2 — Supplementary Material 2 [file 12909_2025_7213_MOESM2_ESM.pdf]

Appendix B: Post test questionnaire

Post-test questionnaire for the further development and improvement of continuing education and training programs for specialists in plastic-aesthetic surgery

Socio-demographic issues and occupational characteristics

- Age in years _______ *(free text)*
- Gender *(Single Choice)*
  O female
  O male
  O divers
- Activity performed ____________ *(free text)*

Questions about the Wrinkle Course

- How satisfied were you overall with the wrinkle course?
  O extremely satisfied O very satisfied O somewhat satisfied O a little dissatisfied O very dissatisfied O extremely dissatisfied

- How satisfied were you with the course content?
- O extremely satisfied O very satisfied O somewhat satisfied O a little dissatisfied O very dissatisfied O extremely dissatisfied

- How satisfied were you with the structure of the course?
- O extremely satisfied O very satisfied O somewhat satisfied O a little dissatisfied O very dissatisfied O extremely dissatisfied

- How satisfied were you with the frequency of online meetings?
- O extremely satisfied O very satisfied O somewhat satisfied O a little dissatisfied O very dissatisfied O extremely dissatisfied

- How satisfied were you with the course material that was provided for self-study?
- O extremely satisfied O very satisfied O somewhat satisfied O a little dissatisfied O very dissatisfied O extremely dissatisfied

- How satisfied were you with the learning platform Moodle (PAAU Academy) in terms of learning support?
- O extremely satisfied O very satisfied O somewhat satisfied O a little dissatisfied O very dissatisfied O extremely dissatisfied

- How satisfied were you with the learning platform Moodle (PAAU Academy) in terms of social aspects such as the formation of a community of practice?
- O extremely satisfied O very satisfied O somewhat satisfied O a little dissatisfied O very dissatisfied O extremely dissatisfied

- How satisfied are you overall with the learning progress after the wrinkle course?
- O extremely satisfied O very satisfied O somewhat satisfied O a little dissatisfied O very dissatisfied O extremely dissatisfied

- How did you feel about the length of the wrinkle course?

O too short O just right O too long

- From your point of view, has a community of practice formed that still gives the opportunity for mutual exchange after the end of the course?
  O yes O no

- Have you already carried out wrinkle injections with botulinum toxin A after completing the course?
  O yes O no

- Did the wrinkle course help you to feel more confident when carrying out the injection with botulinum toxin A?
  O yes, because *(free text)*
  O no, because *(free text)*
  O not applicable

- Have you already carried out wrinkle injections with dermal filler (e.g. hyaluronic acid) after completing the course?
  O yes O no

- Did the wrinkle course help you to feel more confident when performing the injection with dermal fillers (e.g. hyaluronic acid)?
  O yes, because *(free text)*
  O no, because *(free text)*
  O not applicable

Questions about the learning objectives

- Please indicate how you assess your level of knowledge on the following topics (very good (1); good (2); satisfactory (3); sufficient (4); poor/poor (5); inadequate/very bad (6)):

	(1)	(2)	(3)	(4)	(5)	(6)
Anatomy of the face						
Aesthetics of a beautiful and natural face						
Facial aging						
Legal aspects, hygiene and liability for medical treatment						
Education and documentation (incl. photo documentation) of patients						
Patient communication						
Preparation of a treatment plan						
Risks of wrinkle injections						
Selection of suitable injection products (botulinum toxin type A or hyaluronic acid)						

Appendix B: Post test questionnaire

Indication and contraindication of wrinkle treatments						
Injection system and injection technique for the injection of wrinkles						
Complication management in wrinkle treatment						
Pre- and aftercare for wrinkle treatment						
