# Supplementary material for: The effectiveness of further education and training programs for plastic and aesthetic surgeons: an evaluation according to Kirkpatrick levels 1–3
Source: BMC Med Educ. 2025 Apr 30;25:636. doi: 10.1186/s12909-025-07213-8 (PMC12042626; doi:10.1186/s12909-025-07213-8)
Supplement: Supplementary file 3 — Supplementary Material 3 [file 12909_2025_7213_MOESM3_ESM.pdf]

Appendix C: SRQR-Checklist

SRQR-Checklist Evaluation Kirkpatrick Level 1-3

No.	SRQR criterion	Description	Implementation in this study
1	Title and Abstract	Is the title clear and describes the nature of the study?	The effectiveness of further education and training programs for plastic and aesthetic surgeons: An evaluation according to Kirkpatrick levels 1-3
2	Problem definition and objective	Is the research problem clearly described?	Kirkpatrick Level 1-3: The main objective of the study is to evaluate the effectiveness of modern training and further education using the example of plastic-aesthetic surgery. This involves initially examining the reactions of participants to the training and further education, how knowledge acquisition develops, and whether the acquired knowledge was applied in practice after the end of the training and further education.
3	Theoretical context	Is there a clear theoretical model or conceptual basis?	Kirkpatrick Model Level 1-3
4	Methods	Which methods were used?	Pre and Post Questionnaire for participants of the first wrinkle course
5	Participants and Setting	How were the participants selected?	All participants from the first wrinkle course were chosen
6	Data collection	How was the data collected?	Questionnaires were analyzed using statistical methods; a content analysis was used for the free text responses
7	Relationship Participants / Researcher	Was there any reflection on how the relationship between researchers and participants might influence the data?	There was no relationship between the researchers and the participants
8	Sampling Strategy	How was the sample defined and why was this method chosen?	At that time there was only the first wrinkle course, so all participants were invented to take part in the questionnaire surveys
9	Ethic Aspects	Was an ethical authorization obtained?	An ethics vote of the University Witten/Herdecke is available. Written consent was obtained from the participants in accordance with the GDPR.
10	Data Analysis	How was the qualitative data analyzed? How was the quantitative data analyzed?	Content analysis by Mayring; statistical methods such as mean values, standard deviation, variance and Cohan´s d
11	Integration of Data	Were several data sources or perspectives used?	In addition to the questionnaires, data from a learning management system was analyzed
12	Reliability of Analysis	What measures have been taken to ensure credibility?	Qualitative Data: Intercoder-Reliability through two independent encoders.<br>Quantitative Data: different statistical methods

Appendix C: SRQR-Checklist

No.	SRQR criterion	Description	Implementation in this study
**13**	Reflexivity	Was the role of the researcher reflected?	The researchers have reflected their role, the perspectives and possible influencing factors on the research process and the interpretations of the results.
**14**	Results	Are the results clearly presented and documented with quotes?	Quotes from interviews illustrate the core statements and current literature was also used
**15**	Discussion of Results	How are the results compared with existing studies?	The results were compared with previous studies on the effectiveness of education and training programs an elearning
**16**	Interpretation and Conclusion	Are the most important findings clearly summarized?	The most important findings were summarized for levels 1 to 3 and correlated with each other.
**17**	Transferability	How can the results be transferred to other contexts?	Results are applicable to similar organizations with comparable education and training courses
**18**	Limitations	Are possible limitations of the study mentioned?	The results cannot be generalized due to the sample size. In the study are the limitations mentioned
**19**	Link between theory and results	Are the results linked to the theoretical framework?	Level 1-3 of Kirkpatrick's model were examined
**20**	Relevance for practice	What practical implications do the results have?	Optimization of the wrinkle course based on the results of the behavioral change. Hands-on Courses for practical conduction of Injections should be implemented
**21**	Complete Reporting	Are all SRQR-criteria documented in the study?	All SRQR-criteria were explained in the study and are included in this appendix.
